# Understanding family planning decision-making: perspectives of providers and community stakeholders from Istanbul, Turkey

**DOI:** 10.1186/s12905-021-01490-3

**Published:** 2021-10-09

**Authors:** Duygu Karadon, Yilmaz Esmer, Bahar Ayca Okcuoglu, Sebahat Kurutas, Simay Sevval Baykal, Sarah Huber-Krum, David Canning, Iqbal Shah

**Affiliations:** 1 Bahcesehir Universiy, Istanbul, Turkey; 2 Harvard T.H. Chan School of Public Health, Boston, MA USA

**Keywords:** Family planning, Decision-making, Health decisions, Qualitative data, Turkey

## Abstract

**Background:**

A number of factors may determine family planning decisions; however, some may be dependent on the social and cultural context. To understand these factors, we conducted a qualitative study with family planning providers and community stakeholders in a diverse, low-income neighborhood of Istanbul, Turkey.

**Methods:**

We used purposeful sampling to recruit 16 respondents (eight family planning service providers and eight community stakeholders) based on their potential role and influence on matters related to sexual and reproductive health issues. Interviews were audio-recorded with participants' permission and subsequently transcribed in Turkish and translated into English for analysis. We applied a multi-stage analytical strategy, following the principles of the constant comparative method to develop a codebook and identify key themes.

**Results:**

Results indicate that family planning decision-making—that is, decision on whether or not to avoid a pregnancy—is largely considered a women’s issue although men do not actively object to family planning or play a passive role in actual use of methods. Many respondents indicated that women generally prefer to use family planning methods that do not have side-effects and are convenient to use. Although women trust healthcare providers and the information that they receive from them, they prefer to obtain contraceptive advice from friends and family members. Additionally, attitude of men toward childbearing, fertility desires, characteristics of providers, and religious beliefs of the couple exert considerable influence on family planning decisions.

**Conclusions:**

Numerous factors influence family planning decision-making in Turkey. Women have a strong preference for traditional methods compared to modern contraceptives. Additionally, religious factors play a leading role in the choice of the particular method, such as withdrawal. Besides, there is a lack of men’s involvement in family planning decision-making. Public health interventions should focus on incorporating men into their efforts and understanding how providers can better provide information to women about contraception.

**Supplementary Information:**

The online version contains supplementary material available at 10.1186/s12905-021-01490-3.

## Background

There is considerable literature on the decision-making process related to fertility, and various factors have been proposed as predictors of family planning decision-making. Women’s characteristics, such as age, parity, level of education, level of income, occupation, and work status are the most frequently cited factors [1, 2]. Additionally, previous studies have analyzed diverse factors that influence family planning decision-making within the family, such as power relations [3] and dominance of male partners [2, 4]. Various studies in Turkey have found that many men are motivated to use family planning and would like to share responsibility for family planning decision-making (to use or not use any family planning method) [5, 6]. However, there is also a tendency to view family planning as “woman's domain,” which refers to deciding whether to avoid pregnancy or not [7].

We would like to emphasize that cultural values also play an important role in impacting the use of family planning. Among these cultural factors, perhaps religious values top our list. Previous studies have also included ethnicity, male preference, traditional family values as well as the economic value of children as potential causal factors in determining family planning decisions. The present study aims at identifying significant contextual factors that are likely to influence use of family planning such as socio-cultural and religious norms.

In the 1960s, Turkey adopted a national family planning policy that advocated the use of both traditional and modern contraceptive methods (i.e., sterilization, intrauterine devices (IUDs), implants, injectables, pills, condoms, emergency contraception, lactational amenorrhea (LAM), and standard days method), and expanded access to contraception through health clinics. According to the 2018 Turkey Demographic and Health Survey, women are very knowledgeable about contraception: 97% of all Turkish women know at least one method of contraception [8]. Further, almost half of married women use a modern contraceptive method [8]. The most commonly used modern methods are male condoms (19%), IUDs (14%), and female sterilization (10%) [8]. However, while the use of modern contraceptives increased steadily in the 1980s and 1990s, the prevalence rate has stagnated since the 2000s. Further, a sizable proportion of women continue to rely on traditional methods of family planning, such as withdrawal [8].

The dominant (almost exclusive) religion in Turkey is Islam. The government, which has been in power since 2002, actively promotes policies that encourage high fertility and discourage contraception and abortion. The Turkish Ministry of Health is responsible for designing and implementing health policies and overseeing all private and public healthcare services in the country. All residents of Turkey who are registered with the Sosyal Güvenlik Kurumu (SGK)^1^ can receive free medical treatment in hospitals contracted by the agency. The services are provided by government hospitals, Aile Sağlığı Merkezi (ASM),^2^ Ana Çocuk Sağlığı ve Aile Planlama Merkezi (AÇSAP),^3^ maternity, and children’s hospitals, training and research hospitals, university hospitals, private hospitals, and private polyclinics. Family planning and abortion services are provided both in public, and private sectors, and modern methods may be accessed for free in government-funded primary health care units and hospitals or from pharmacies and private practitioners for a fee [9]. In general, most women and couples obtain modern contraception from public sector sources, and pharmacies are the leading source of oral contraceptives and male condoms [8]. Women and men can also purchase emergency contraception, hormonal and copper IUDs, three-month contraceptive injections (Depo-Provera), and one-month contraceptive injections (Mesigyna) from pharmacies. IUDs cannot be inserted at pharmacies but are taken to health facilities to be inserted. Male condoms can also be purchased from markets and beauty shops.

The Turkish national curriculum does not provide sex education and the subject is rarely discussed in schools [10]. Since there is no formal education on reproductive health, most people are informed about family planning though friends, relatives as well as printed or social media. Basic information, education, and communication materials about contraception are provided by health facilities.

This study aims to delineate the factors that influence family planning decision-making processes from the perspectives of community stakeholders such as prayer group leaders, parent-teacher association members, and family planning service providers. We attempt to understand and explain these factors within the context of social and political tensions in Turkey most important of which are ethnic and secular-religious cleavages.

## Methods

### Study procedures

We used purposive sampling [11, 12] to interview eight family planning service providers and eight community stakeholders in Bagcilar, Istanbul. Our sample includes fifteen females and one male participant. We determined the number of interviews based on the principles of theoretical saturation (i.e., the criterion for judging when to terminate interviewing at the point when no new information was being generated [13].

Bagcilar is one of the largest districts in Turkey with a population of 745,125 in 2019 [14]. We sampled key informants from different professional backgrounds, with different social status within their respective communities, and based on their role in influencing reproductive health. This enabled us to understand broader community and provider perspectives about women’s health concerns. In-depth interviews were conducted between April and May 2019.

We partnered with a local research firm that had extensive experience in conducting qualitative studies in the area. The research firm and research team generated a list of potential community stakeholders (such as members of local government, religious leaders, women’s groups, and community groups) and the research team made visits to the study area to organize the interviews. We identified service providers from public and private hospitals that offered family planning services in the study area, from a health facility assessment that we conducted less than six months prior. To map the availability of and access to family planning and abortion services, we conducted a facility survey in public and private facilitates that provided reproductive health services in the study area. The facility survey captured data on service availability and facility readiness (including staffing, hours of operation, and payment of user fees), services provided (including counseling, physical examination and contraceptive, and abortion methods), and commodity supplies. These were supplemented with in-depth interviews with key informants. The research firm used separate standardized scripts to recruit family planning providers and community stakeholders. The recruitment script included details about the study, its aim, and contact information for the principal investigators. The research firm scheduled a time for interview with providers and community stakeholders who were willing to participate in the study.

All respondents spoke Turkish and interviews were conducted by a trained Turkish female interviewer who was employed by the research firm. The interviewer had a university degree and was employed as a fieldwork director by the local research firm at the time of the interview. After a refresher training session about principles and techniques of qualitative research, ethics and confidentiality, and role-playing exercises with a supervisor, the interviewer piloted two different semi-structured interview guides (see selected questions in Table 1) (one interview with a family planning service provider and one interview with a community stakeholder). The interview guides were developed for this study in English and translated into Turkish (see Additional files 1 and 2). A randomly selected sample (approximately 5%) of the transcripts were back-translated and reviewed by the research team to ensure that translations were consistent and of high quality. The service provider interview guide included several topics related to accessing family planning, factors influencing decision to use contraception, and barriers to and facilitators of family planning use in the community. The community stakeholder interviewer guide captured information on socio-cultural beliefs influencing community preferences and attitudes regarding family planning. Topics were related to the availability and accessibility of contraceptives, the demand for contraception and abortion services, the influence of attitudes and beliefs on contraception and abortion accessibility, decision-making, and behavior of women regarding gender norms and decision-making between couples. The Turkish version of the interview guide was amended based on questions and feedback obtained during training and pilot test.

**Table 1:** Selected questions from semi-structured interview guides

Family planning service providers	Availability and quality of family planning and abortion services	Can you tell me about the family planning services available at this facility?
		Can you tell me about the demand for family planning and abortion services in this area?
		What do you think most influences women’s desire to use modern family planning methods?
	Experiences providing reproductive health services at this facility	In general, how do you feel about providing family planning services?
		Can you think of any situations in the past in which you were uncomfortable providing family planning services?
Community stakeholders	Availability and demand for family planning and abortion services	Can you tell me about the demand for family planning that you observe in your community?
		What does the local community think about family planning and abortion in general?
		What are the local beliefs or customs that influence women’s use of family planning, perceptions of specific methods’ safety and effectiveness?

All participants received written information about the study and provided oral consent to participate. We did not collect any identifying information from participants. Face-to-face interviews were conducted in a private space (i.e., private rooms at the facilities for family planning service providers and community stakeholders’ homes), and audio recorded with permission from the participants. The interviewer took field notes during the interview. On average, interviews lasted approximately one hour. After interviews were completed, the research team transcribed each interview in Turkish and then translated it into English for coding and analysis. Transcripts were double-coded by the research team to ensure accuracy. We did not share transcripts with participants. Before data collection we received ethical approval from the Boards of Harvard School of Public Health and Bahcesehir University.

### Analysis

We used ATLAS.ti (Version 8.0, Scientific Software Development, Berlin) to manage and analyze the data. We further used an inductive, thematic analytical approach, guided by the principles of the constant comparative method to identify key themes arising from the data [12]. First, four researchers reviewed eight transcripts and developed an initial list of codes and general themes (see Additional file 3). Specifically, we used in vivo coding in ATLAS.ti to code participants’ spoken words and used their own words as codes. For example, one participant commented, “*One of my clients said that she would not use birth control pills because it was a sin.”* The final title of the code became “sin” and similar statements referring to abortion as a sin were grouped together to create the theme. Next, four members of the study team read two transcripts aloud together and open-coded all text, in line with the principles of open coding and an inductive approach [12]. We reviewed all codes together (more than 200 codes), merging similar codes and grouping codes into themes and sub-themes. Next, once all major themes and sub-themes were agreed upon, we generated a final codebook, which included 51 sub-codes in six main coding groups, including demographics, family planning, abortion, socially-oriented perspectives, quality of services, and family planning programs. The study team double coded all transcripts. Two members of the study team were assigned to each interview in order to enhance the quality of the analysis.

Several key themes emerged from the data related to family planning decision-making. All themes were identified by two members of the study team. We decided to characterize emerging dominant themes related to most frequently discussed topics across all interviews.

## Results

### Participants’ profile

Background characteristics of participants are shown in Table 2. There were six physicians/gynecologists and two midwives in the group of family planning providers. The service providers in our sample had been providing family planning services for between one and 22 years. Regarding community stakeholders, two were associated with Ak Parti—the religiously conservative, ruling political party- as members^4^ and representatives,^5^ two were local parent-teacher association members, one was a neighborhood representative’s assistant and another was a member of a local prayer group. Additionally, there was a pharmacist and a pharmacist’s assistant in the group of community stakeholders. The pharmacist and the pharmacist’s assistant were assigned to the community stakeholder group since they frequently provided informal advice to women about contraceptive use and other reproductive health-related topics.

**Table 2:** Background characteristics of key informants

Participant number	Type	Sex	Current position/community role	Length in current position	Length providing family planning services
01	Community stakeholder	Female	Ak Parti member and Parent-teacher association member	–	–
02	Community stakeholder	Female	Prayer group leader	–	–
03	Community stakeholder	Female	Pharmacist	36 years	–
04	Community stakeholder	Female	Pharmacy assistant	20 years	–
05	Community stakeholder	Female	Neighborhood representative’s assistant	–	–
06	Family planning provider	Female	Physician	8.5 years	8.5 years
07	Family planning provider	Female	Midwife	14 years	14 years
08	Community stakeholder	Female	Parent-teacher association member	–	–
09	Family planning provider	Male	Gynecologist	20 years	20 years
10	Family planning provider	Female	Gynecologist	14 years	22 years
11	Community stakeholder	Female	Ak Parti neighborhood representative	–	–
12	Community stakeholder	Female	Parent-teacher association member	–	–
13	Family planning provider	Female	Gynecologist	1 year	1 year
14	Family planning provider	Female	Midwife	10 years	10 years
15	Family planning provider	Female	Gynecologist	4 months	7 years
16	Family planning provider	Female	Gynecologist	17 years	17 years

### Key themes

We wanted to understand family planning decision-making process in relation to decisions about whether to avoid pregnancy or not. Three main themes identified by the study team emerged from the transcripts, including the decision-making process, the role of male partners, and the role of religious beliefs on reproductive health decisions, that provide insight into how women and couples decide to use contraception, how they learn about contraception, and the types of contraceptives women and couples prefer (Table 3). In general, we found that there was considerable demand for modern contraceptives among women. The majority of respondents mentioned the increasing awareness about modern contraceptive methods, most notably young women wishing to delay or space childbearing and women who wish to limit births once they achieve their ideal family size. Providers’ narratives implied that they are supportive of these growing modern contraceptive trends and actively encouraged young women to take actions to meet their reproductive needs.

**Table 3:** Key themes related to family planning decision-making

Key themes	Definition	Example of direct quotations
Decision making process	Decision-making involvement, freedom to choose methods or inability to choose and accessibility	*Everyone has a different choice; for example, I use the pills, but I put on a lot of weight. So, if I had something else, I wouldn’t prefer the pills*. (Interviewee 2, Community stakeholder)
Role of male partners	Demand for contraception, childbearing responsibilities and men’s desire to have more children	*[Men] don’t care about having a lot of children as they don’t have to look after them…[men] want to make children* (Interviewee 5, Community stakeholder)
Role of religious beliefs	Barriers to using or accessing family planning services related to religion	*As far as I heard, it [family planning] is a sin* (Interviewee 12, Community stakeholder)

#### Decision-making process: preferences and access

Respondents differed in what they perceived as the most preferred contraceptive method for women. While they discussed a variety of modern contraceptive methods used by women in their communities, many agreed that traditional methods, such as withdrawal or periodic abstinence, were preferred. They frequently believed these traditional methods to be more *effective* than other modern methods, and also explained that women prefer these methods to avoid side effects and also for convenience in use. A gynecologist who had been serving in this position for four months said:

*If you leave it to the clients, they will still use the withdrawal method. It does not matter if they are educated or not. Nearly 70% of them still use the withdrawal method… They think it is safe. They say they have been using it for five years and nothing happened, so they continue [to use it]*. (Interviewee 15, Family planning provider)

Most respondents agreed that the use of contraception is a woman’s decision. A parent-teacher association member noted that “…*women have to think about [birth control] as they are the ones who take care of the children. So the women make the decisions.”* (Interviewee 8, Community stakeholder). Moreover, another participant summarized the situation with the following comment: “*Because they [women] don’t want to get pregnant. It is always the women who endure the hardships of pregnancy, so they make the decisions”* (Interviewee 11, Community stakeholder). Although most respondents agreed that women are more likely than men to be involved in the choice of a preferred contraceptive method, decision-making within a family is multi-layered. Some respondents reported that mothers-in-law and fathers-in-law are also important actors who exert an influence on family planning matters. A physician who had been providing family planning services for approximately nine years explained:

*I had a few clients whose mothers-in-law wanted their daughters-in-law to have more children. And this affects the spouses or the husbands and they think about having another child. As they live together, the mother-in-law or even the father-in-law influences [their decisions to have another child]*. (Interviewee 6, Family planning provider)

All participants reported that modern contraceptive methods are widely available and easy to access from health care centers and pharmacies. The majority of respondents (both providers and community stakeholders) reported that women trust and respect family planning service providers. Nevertheless, with regard to obtaining information, women trust the contraceptive experience of other people like their friends and family members and therefore mostly rely on second hand information. A community stakeholder commented:

*First, they [women] talk among themselves. For example, she asks me how I manage birth control, how I prevent pregnancy. I say that I use the pill or injections or that my husband uses a method. She says that if it is good, she will do it too. Then she goes to the health center to ask the nurses…It is the culture of the women here, nothing else. It is better for them to hear it instead of searching and learning, I think*. (Interviewee 1, Community stakeholder)

A few participants discussed the influence that the characteristics of providers can have on decision-making. The narratives suggested that decision-making is influenced by accessibility and quality of services. One community stakeholder said:

*If the doctor is male, women are shy, but just a little…Their husbands don’t let them [go]. They say “If the doctor is male, you can’t go… When we go there again with our husbands, and the doctor will say that I have been here before. Then I will have problems with my husband” they say*. (Interviewee 1, Community stakeholder)

Most participants did not report difficulties with accessing contraception for any particular group of women, and they agreed that unmarried women and adolescent girls can access modern contraception. A few reported that modern contraceptive methods are available, but it is difficult for single women to obtain them, which is an indication of barriers to access among this sub-group of women.

*As far as I know, single persons wouldn’t get them from somebody they know [meaning a provider, pharmacist, or friend]. It is easily accessible, but the social pressure is serious. So, it is easily accessible, but it is hard to get*. (Interviewee 3, Community stakeholder)

Our findings suggest that there is no single explanation for family planning decisions among women in the study setting. Various factors influence family planning decisions, and factors such as the source of information, characteristics of service provider, and marital status play a role.

#### Role of male partners

Most respondents stated that demand for modern contraceptive services is stronger among women compared to men. The majority of respondents reported that men do not favor modern contraceptive use, but do not actively object to using them. It was evident from participants’ narratives that family planning decisions remain a “woman’s domain”—that is, it is women who typically decide whether to avoid pregnancy or not. Additionally, family planning service providers reported that men have very limited involvement with pregnancy planning and fertility decisions and that women often do not trust men to be involved in such decisions.

*Men are not trusted to be involved with family planning by women. Men are fine with [women’s decisions] …I think this responsibility is given to the women in Turkey. Men do not care about it much*. (Interviewee 15, Family planning provider)

A gynecologist who has provided family planning services for 14 years said:

*…men have birth control methods such as withdrawal and condoms but generally the women come here to consult about the methods. But a lot of men use birth control too. When the women use IUD or the pill and experience side-effects, I think the men understand and they resort to methods such as withdrawal and condoms*. (Interviewee 10, Family planning provider)

Participants reported that men are more likely to desire more children compared to women, but the burden of childrearing falls on women. A local midwife who had provided services for ten years in the community explained:

*When [women] bear a child, most husbands do not help with childcare. It is as if the child belongs only to the mother; supposedly, he is the father. When the child is sick, the mother takes care of him/her; and when the mother is sick, the father cannot take care of the child… Men generally say that they are unable to take care of children. So, women want birth control methods to avoid consecutive births*. (Interviewee 14, Family planning provider)

A local pharmacist who has been in that position for 36 years also indicated that men desire to have more children than their wives. She said:

*…especially the husbands want more children, so the women sometimes get these [family planning methods] without telling their husbands*. (Interviewee 3, Community stakeholder)

#### The role of religious beliefs

Participants reported few barriers to contraception, and the narratives suggest relatively few reasons for non-use. However, a frequent theme was the importance of religious beliefs on reproductive health decisions. A few participants reported that women believe that modern contraception, in general, or use of certain methods in particular, are sinful behavior. A gynecologist who had been in that position for 20 years said:

*…our religious belief is against it; according to our faith, family planning is forbidden. What can you do with this person? He/she wouldn’t do it even if it were free*. (Interviewee 9,^6^ Family planning provider)

Further, a parent-teacher association member and a gynecologist reported that:

*Some spouses consider [birth control] to be a sin. We hear it from our friends*… Interviewee 8, Community stakeholder)

* Actually, there is prejudice against most of the birth control methods in our society… Modern contraception is considered a sin. They [referring to the people in the community] do not want birth control. Women do not want IUD. They use the withdrawal method*. (Interviewee 13, Family planning provider)

Additionally, beliefs about the moral status of contraception seem to be influenced by women’s social networks. A pharmacist described the effects of shared beliefs around contraceptive decision-making, thus:

*One of my clients said that she would not use birth control pills because it was a sin. A couple of months later, she got pregnant and had to have an abortion. I asked her who had recommended it; it turned out to be someone I knew. Then I called that person and said “Why are you misinforming people?” She told me that it was a sin. I told her “Isn’t abortion a sin? She had to have an abortion.” She said that it was not alive until it was three months old. I told her “Look, you don’t have the knowledge about it but you have opinions. You are misinforming people and playing with their lives. A lifeless thing does not grow; it is alive since the first moment that sperm fertilizes the egg. Do not misinform people, please. Send the people to the health centers or doctors but don’t misinform them.” She was offended but I think that the conversation was effective. (Interviewee 3, Community stakeholder)*

The narratives suggest that there is contradiction between faith and behavior. In particular, women think that contraception could be against the will of God, but act in accordance with the dictates of modern life.

## Discussion

The findings from this study highlight the major factors that influence family planning decision-making. According to the 2018 Turkey Demographic and Health Survey, 99.5 percent of married women of reproductive age know at least one method of contraception [8]. Our results are consistent with the existing literature which shows that contraceptive methods (either modern or traditional method) are widely known in the community. Thus, a key finding from the study is that women, and particularly married women, are aware of at least one method of contraception. Therefore, high levels of knowledge of contraceptives provide opportunities for programs to address barriers that could hinder translation of such knowledge into practice.

We found that, according to the perceptions of key informants, traditional methods were preferred over modern methods, and most respondents explained that women prefer traditional methods mostly due to the absence of side effects and ease of use. There is widespread perception that modern methods might have undesired side effects. Additionally, there are religious reasons such as couples’ consideration of natural, easy use the method with more minor side effects for traditional methods being the most preferred methods. According to Cebeci et al., however, even religious beliefs should not be identified as the dominant barrier to contraceptives; they rather affect the choice of particular methods such as withdrawal [7]. The effect of religious beliefs on contraceptive choice may be the reason why couples continue to rely on traditional methods. There is, however, a need for studies to better understand the motivations for preference for traditional methods in the study setting and how women could be supported to ensure that such methods meet their reproductive needs.

Participants reported that family planning is a “women’s domain” although sometimes other family members, such as mothers-in-law and fathers-in-law, may influence decision-making. A study among married individuals in Umraniye which is another district of Istanbul also found that family planning decision-making was perceived as a “women’s issue” by male partners [7]. Yet, decision-making is not limited to women and women’s partners; family members are also involved in their contraceptive choices. These patterns underscore a need for a better understanding of intra-family relations and opportunities that such relations provide for supporting women in the study setting to realize their reproductive goals.

Our findings show that although women trust family planning providers on contraceptive issues, they have more confidence in the previous family planning experiences of other people like their friends, neighbors, or relatives. This underscores the significance of women’s social networks as a source of information as well as a determinant of behavior. As Yee and Simon found, women identified their social networks as one of the most influential factors in the family planning decision-making process, especially about side-effects, safety, and effectiveness, and most of them considered that information more reliable than other sources of information [15]. Husbands, however, do not tend to share information about contraception with one another. Thus, husbands may look to their wives to receive accurate and reliable information about contraception [16]. Understanding how women’s and men’s social networks influence contraceptive use in this setting may be key to increasing contraceptive use among women who do not want a pregnancy. Intervention studies might also consider leveraging women’s social networks to provide education about contraception (e.g., peer educators or women’s groups).

Related to the accessibility and quality of services that influence decision-making, our findings show that women prefer female to male physicians and consultants in matters related to contraception. In addition, some of the community stakeholders reported prejudice in accessibility to contraceptive methods against unmarried women. Pharmacies provide male condoms, pills, and emergency contraception without a written prescription in Turkey. The pharmacy sector provides more than 45% of the male-condom and pills [8]. Many unmarried women find it more convenient to obtain contraceptive supplies from pharmacies, despite contraception not being free at pharmacies. This is likely because many single women prefer to avoid social pressure in healthcare facilities and fear being ostracized for engaging in what is regarded as illegitimate sex. The finding that many women in the study setting prefer obtaining contraceptives from pharmacies suggests a need for improving the capacity of pharmacists to provide contraceptive information and counseling to clients.

Various studies in Turkey have found that a variety of perspectives need to be taken into account to fully understand family planning decision-making processes. On the one hand, men report that family planning is a shared responsibility [6], and that pregnancy planning should be done jointly between partners [17] which is consistent with existing evidence showing that male involvement and shared decision-making is a key element of reproductive decisions [5, 18]. On the other hand, several studies show that men and women are not resistant to contraception, although women are perceived to be the ones making family planning decisions [7, 19]. Our findings show that men are not much involved in family planning decision-making and it is often women who decide whether to avoid pregnancy or not. While some respondents suggest that men might be opposed to contraception, the majority reported that men were simply indifferent. Additionally, lack of men’s involvement likely stems from pro-natalist views. The findings suggest a need for a better understanding of couple-level contraceptive decision-making and how best to engage men in supporting women’s reproductive needs.

Studies show that various factors influence fertility decisions, including the number of living children [20, 21], level of education of parents and especially of female partners [22], and socio-cultural norms and religious attitudes [17]. However, men, in almost every setting, desire more children than women [17, 23]. In general, both family planning service providers and community stakeholders in our study reported that men desire more children compared to women. However, the burden of childrearing falls on women, which reflects gender roles in the family. Men’s desire for children could be associated with a need to continue the family line and enhance their social value [24], making sense in terms of the social value of having a child primarily for men [25]. This a further indication of the need for understanding the perspectives of men in the study setting and how best to involve them in supporting women’s reproductive needs.

Our findings showed that women placed greater importance on religious beliefs although in practice, such beliefs did not have a direct influence on decisions regarding family planning. Although women believed that contraception could be against the will of God, this did not stop them from using the methods. This is consistent with findings from another qualitative study which showed that religious beliefs were not barriers to contraception, but such beliefs influenced the choice of methods [7]. Religion does not often dissuade women and men from wanting small families, but instead of using the most effective methods, they instead rely on methods that they perceive to be in alignment with religious beliefs or methods that are not as bad as others. Although most respondents in our study reported that contraception is perceived as a sin, women still used methods. It is possible that religious values may encourage the use of traditional methods, such as withdrawal, that have a long and historical tradition of being used in this setting. Cebeci and colleagues found that, in addition to people’s consideration of withdrawal as a natural, easy to use method with less side effects compared to modern methods, some considered it the method encouraged by Prophet Muhammad, which indicates that modern methods are perceived as harmful [7]. The findings underscore a need for family programs in the study setting to incorporate empowerment principles in client counseling in order to address misconceptions about modern contraceptives influenced by religious beliefs.

Our findings may be influenced by the manner in which participants were selected. In particular, community stakeholders and service providers were purposively selected based on their familiarity with women’s reproductive health-related topics, including family planning, and the sample included only one male participant. All interviews were conducted in Turkish and translated into English for analysis. Although some meanings could be lost in the process, a small sample of the transcripts were back-translated to determine the extent of such loss. There was no loss in meanings due to translation from one language to another. Additionally, all interviews were conducted in a private space to reduce the risk of social desirability bias. By its very nature, our sample has limited external validity which prevents us from making inferences about patterns in the study setting or the country as a whole. Although our findings, based on a limited purposive sample with key informants, are consistent with the findings of other studies using larger samples with more diverse groups of women, further qualitative research with representative samples of reproductive age women is needed to determine the extent to which our findings are consistent with the prevailing patterns in the country as a whole.

## Conclusion

Our study sheds light on the factors that play a role in women’s contraceptive decisions in Turkey, a country with a strong national family planning policy but characterized by political-religious differences in beliefs about use of family planning. Our first take is that women (as well as couples) have a strong preference for traditional methods and particularly withdrawal. Religious factors in particular and socially conservative values in general play an important role in the choice of method. However, it should also be noted that the strong preference for traditional methods is a more general phenomenon that is not limited to the prevalence of religious and conservative values.

Second, in most cases, men play a minimal, if any, in family planning decisions. This is of both practical and academic interest especially in a male-dominant culture. From a policy viewpoint it points out to the need of educating not only women but also men about the availability, advantages, disadvantages and possible risk of available methods.

Third and last, the link between values and family planning decisions at all levels seems to be evident and this relationship deserves further investigation.

## Supplementary Information

**Additional file 1:** Key Informant Interview Guide-Community Stakeholders.

**Additional file 2:** Key Informant Interview Guide-Family Planning Service Providers.

**Additional file 3:** Coding tree.

## Data Availability

Anonymized data can be availed upon reasonable request to the first author.

## Abbreviations

IUDs: intrauterine devices
LAM: lactational amenorrhea
SGK: Sosyal Güvenlik Kurumu
ASM: Aile Sağlığı Merkezi
AÇSAP: Ana Çocuk Sağlığı ve Aile Planlama Merkezi

## Footnotes

1 Social Security Institution.

2 Family Health Centers.

3 Maternal and Child Health and Family Planning Centers.

4 AK Parti member is also member of parent-teacher association.

5 They are not politicians, but they act as liaisons between community members and the political party.

6 Male participant.
