# Supplementary material for: Understanding family planning decision-making: perspectives of providers and community stakeholders from Istanbul, Turkey
Source: BMC Womens Health. 2021 Oct 9;21:357. doi: 10.1186/s12905-021-01490-3 (PMC8502330; doi:10.1186/s12905-021-01490-3)
Supplement: Supplementary file 1 — Additional file 1: Key Informant Interview Guide-Community Stakeholders. [file 12905_2021_1490_MOESM1_ESM.docx]

# **Willows Impact Evaluation**

# Key Informant Interview Guide – Community Stakeholders

Interview date**:** ____ / ____ / ______

(day / mo. / year)

Interview start time: ____ ____ : ____ ____ (AM / PM)

Interview location: __________________________________

**Introduction**

As you may know, Willows International has been working in your area over the past [*insert project duration*]. We are conducting a study to understand how the Willows project operates in this community. The purpose of this conversation is to learn from you about community preferences and opinions regarding family planning. We would also like to hear about your perspectives on, and experiences with, the Willows project. The input that you provide is critical, as it will help us to understand how the Willows project may be improved in the future.

**Interview Guide**

**Demographics**

1. What is your profession?
2. What is your specific role in the organization/group?
3. How long have you worked in this role?
4. How long have you lived in this community?

**Availability and Demand for Family Planning and Abortion Services**

1. I’d like to learn from you about the availability of family planning services in this community.
   1. Where (or to whom) do most people go to obtain family planning information?
      1. What do you think about this source/these sources?
   2. Where (or to whom) do most people go to obtain family planning methods?
      1. What do you think about this source/these sources?
      2. Who do women trust the most regarding issues related to family planning?
      3. Who do men trust the most regarding issues related to family planning?
2. Can you tell me about the demand for family planning and abortion services that you observe in your community?
   1. Women vs. men?
   2. Young people vs. older people?
3. How does the demand for FP services compare to demand for other reproductive health services (e.g. fertility services, testing for sexually transmitted infections, antenatal/postnatal care, etc.)?
   1. Women vs. men?
   2. Young people vs. older people?
4. What are the most commonly used modern FP methods in this community?
   1. What do people like most about these methods?
   2. Are these modern methods easily accessible/available?
5. What is it like for women to access family planning services in this community?
   1. Probe for supply-side factors (e.g. availability and quality of services, cost, and distance).
   2. Probe for demand-side factors
      1. Household power and gender dynamics: Which family member(s) make decisions about family planning use?
      2. Local beliefs or customs that influence women’s use of family planning; perceptions on the safety and effectiveness of specific methods?
6. What is it like for men to access family planning services in this community?
7. What is it like for the following groups of women to access family planning services?

**[probe: is it easy or difficult for each of the following groups? Why?]**

- 1. Unmarried women or never-married women?
  2. Women with no children?
  3. Married women
  4. Women with only 1 to 2 children?
  5. Unmarried adolescents?

1. Are abortion services available in this community?
   1. If a woman wants to seek abortion services, what is this process like?
   2. In general, how do members of your community feel about abortion?
      1. Young women
      2. Older women
      3. Men
      4. Relatives/in-laws
2. What does the local community think about family planning and abortion in general?

a. Impact of financial situation?

b. Impact of religion? Is it considered a sin?

c. Do you think that women are talking about family planning and abortion at home meetings/ in conversations or not?

**Opinions of FP programs working in this community:**

1. Are you aware of any organizations/programs working on Family Planning initiatives in this community?
   1. Please name the organization(s) and describe their main activities.
   2. What are your impressions of these organizations?
      1. How do you feel about them?
      2. How does your community feel about these organizations/programs?
2. Have you heard of the Willows Project before today?
   1. Probe: Female (or Male) Field Educators who visit women at home to provide FP/RH counseling and information
   2. Probe: Show “tree” logo

**[IF NOT AWARE OF THE WILLOWS PROJECT, SKIP TO END]**

1. To the best of your knowledge, what does the Willows project do?
   1. Types of activities
   2. Goals of the program
2. Have you had any interactions with the Willows project?
3. What are your general impressions of the Willows project?
   1. How does your community feel about the Willows project?
   2. Can you share any feedback you might have received from other community members about the Willows project?

1. Have any community members benefited from the Willows project?
   1. If yes, which community members have benefited?
   2. How have they benefited? What have you observed?
2. In your opinion, what are the strengths of the Willows project?
   1. What is ***most*** useful aspect of the Willows project? Why?
3. In your opinion, what are the weaknesses of the Willows project?
   1. What is ***least*** useful aspect of the Willows project? Why?
   2. Do you have any concerns about the Willows project?
4. If Willows was going to launch in another community in this city, what aspects of the program would you:
   1. Advise them to do differently?
   2. Advise them to continue?

**Closing and Summary**

This is the end of the interview. Is there anything else you would like to discuss or tell me that you have not done so already?

Thank you for your time and cooperation.

**Interview end time:**

**____ ____ : ____ ____ (AM / PM)**
