# Supplementary material for: Understanding family planning decision-making: perspectives of providers and community stakeholders from Istanbul, Turkey
Source: BMC Womens Health. 2021 Oct 9;21:357. doi: 10.1186/s12905-021-01490-3 (PMC8502330; doi:10.1186/s12905-021-01490-3)
Supplement: Supplementary file 2 — Additional file 2: Key Informant Interview Guide-Family Planning Service Providers. [file 12905_2021_1490_MOESM2_ESM.docx]

# **Willows Impact Evaluation**

# Key Informant Interview Guide – Family Planning Service Providers

**Administrative info:**

Interview date**:** ____ / ____ / ______

(day / mo. / year)

Interview start time: ____ ____ : ____ ____ (AM / PM)

Interview location:

**Introduction**

Today we will discuss topics regarding the provision of family planning services in this facility and in this community. I would also like to learn about family planning programs in this area, including the Willows project. The purpose of this conversation is to understand whether and how this facility works with Willows to increase access to family planning, and to understand the barriers and facilitators of family planning use in this community. As a service provider, your perspectives are important and your insights are critical to understanding these relationships and processes.

**Interview Guide**

**Demographics**

1. What is your role at this facility?
2. Do you work at this facility full time?
3. How long have you worked at this facility?
4. How long have you been providing family planning services in this community?
5. Do you live in this community?
   1. If yes, how long have you lived in this community?

**Key Questions**

1. **Availability and Quality of FP/RH Services**
2. I’d like to begin by discussing the issue of quality in family planning services. In your opinion, what would “high quality” family planning services look like?

[**interviewer note: this is a hypothetical question; we want to understand the provider’s definition of the ‘ideal’ scenario, which may differ from the reality in this facility]*

1. Can you tell me about the family planning services available at this facility?
   1. Family planning counselling/education – what topics?
      1. How is this information provided? And by whom?
      2. Format? (Individual, on a needs basis or scheduled group counselling)
   2. Types of family planning methods available
   3. Probe: Are abortion services provided?
      1. To whom? And under what circumstances?
      2. Do women require permission from anyone else to obtain abortion services?
2. What are the most commonly requested modern FP methods in this facility?
   1. What do people like most about these methods?
3. Can you tell me about the demand for family planning and abortion services in this area?
   1. Women vs. men?
   2. Young people vs. older people?
4. What do you think most influences women’s desire to use modern family planning methods?
   1. What about men?
5. How does the demand for family planning and abortion services compare to demand for other reproductive health services (e.g. fertility services, testing for sexually transmitted infections, antenatal/postnatal care, etc.)?
   1. Women vs. men?
   2. Young people vs. older people?
6. Do you feel this facility is equipped to meet women’s family planning needs?
   1. Commodities
   2. Staffing (including training/expertise of staff)
   3. Accessibility
7. **Experiences providing FP/RH services at this facility**
8. In general, how do you feel about providing family planning services?
   1. Can you think of any situations in the past in which you were uncomfortable providing family planning services?
      1. What about services for unmarried or never-married women?
      2. What about services for women without children?
      3. What about women with many children?
      4. What about services for adolescent women who are still in school?
      5. What about women who are accompanied by their male partners?
9. In general, how do you feel about providing abortion services?
10. **Interactions with, and impressions of the Willows Project**
11. Are you aware of Willows International (the Willows project?)
    1. If yes, to your knowledge, how does the Willows project work?
    2. What are the main activities of the Willows project?
    3. Does the Willows project conduct any activities at this facility? If yes, please explain.
       1. Referral system for Willows clients – how does this work?
12. Can you tell me about any interactions that you or your staff have had with the Willows project?
    1. To what extent does the Willows project liaise/interact with health facility management and staff?
    2. Are facility staff willing to collaborate with the Willows project? Why/why not?
13. What are your general impressions of the Willows project?
    1. How do your patients feel about the Willows project?
    2. Have any patients benefited from the Willows project?
       1. If yes, which types/groups of patients have benefited?
       2. How have they benefited? What have you observed?
    3. Can you share any feedback you might have received from other community members about the Willows project?
14. In your opinion, what are the strengths of the Willows project?
    1. What is ***most*** useful aspect of the Willows project? Why?
15. In your opinion, what are the weaknesses of the Willows project?
    1. What is ***least*** useful aspect of the Willows project? Why?
    2. Do you have any concerns about the Willows project?
16. If Willows was going to launch in another community in this city, what would you:
    1. Advise them do differently?
    2. Advise them to continue?
17. Aside from Willows, are there any other family planning programs and/or interventions active in this community?
    1. Probe: Facility vs. community based programs
    2. What are the activities and goals of those programs?
    3. Please describe your impressions of these other community-based programs.
    4. Does this facility have a close interaction with those programs? Please explain.
    5. How are these relationships similar or different to your relationship with the Willows project?

**Closing and Summary**

This is the end of the interview. Is there anything else you would like to discuss or tell me that you have not done so already?

Thank you for your time and cooperation.

Interview end time:

____ ____ : ____ ____ (AM / PM)
